# Swimming through sand: using accelerometers to observe the cryptic, pre-emergence life-stage of sea turtle hatchlings

**DOI:** 10.1098/rspb.2024.1702

**Published:** 2024-10-02

**Authors:** David Dor, David T. Booth, Lisa E. Schwanz

**Affiliations:** ^1^ School of Biological, Earth and Environmental Sciences, University of New South Wales, Sydney, New South Wales, Australia; ^2^ School of Environment, University of Queensland, Brisbane, Queensland, Australia

**Keywords:** green turtle, *Chelonia mydas*, nest, movement behaviour, digging, hatching

## Abstract

Animals that hatch within a subterranean nest, such as turtle hatchlings, expend some of their limited energy reserves digging out through sand or soil to reach the surface. In sea turtles, this emergence process can take the hatchlings 3–7 days. However, we have a poor understanding of this process as it is difficult to observe what is occurring underground. Here, we utilize a novel method to characterize digging-out behaviour: affixing an accelerometer directly to newly hatched green turtles (*Chelonia mydas*) to record movement until nest emergence. Our data revealed that buried hatchlings maintained a head-up orientation but did not move in the expected left and right swaying motion associated with alternating limb crawling. Rather, they moved using dorsal–ventral heaving and pitching as if swimming vertically through the sand to the surface. Movement activity was irregular and brief, interspersed by many short periods of inactivity, mostly lasting less than 10 min. The first 24 h of head-up activity displayed no diel patterns, but the last 24 h prior to emergence involved more intense movement during night-time hours compared with daytime hours. Thus, our results add valuable new insight, and in some cases change previous assumptions, regarding the digging behaviours during the egg-to-emergence life stage in sea turtles.

## 1. Introduction

When sea turtles hatch from their eggs, they are immediately confronted with the arduous task of digging out of their nest. Hatchlings are buried below approximately 4500 cm^3^ of sand, and they must dig vertically 30–80 cm to reach the surface, a process that takes 3–7 days [1,2]. This prodigious task uses both aerobic and anaerobic metabolism within a hypoxic environment [3,4], with the residual yolk being the presumed source of energy [2]. Minimizing the use of this limited source of energy may be critical for maximizing chances of successful dispersal to the offshore ocean where they complete the next phase of their life history.

Unfortunately, the hatching-to-emergence stage in a turtle’s life is cryptic and underexplored, being eclipsed by the large body of literature highlighting the sensitivity of the developing embryos (i.e. the egg stage) to abiotic factors within nests (e.g. temperature and moisture) and quantifying these effects on development, performance and behaviour of hatchlings [5–8]. In addition, the ‘lost years’ between emergence and adulthood in the ocean [9] have garnered considerably more interest than the hatching-to-emergence stage despite the latter’s much greater accessibility to researchers. As a result, most of our perception of hatchlings in the nest is based on anecdote, with limited data from natural nests [10].

Digging out and emergence behaviour were first observed in the 1960s by placing hatchlings in an artificial nest with a glass viewing pane [11,12]. In this setting, hatchlings were described as ‘thrashing’ to the surface predominantly during night-time hours. Moreover, it is believed that hatchling sea turtles dig in coordination and with a loose division of labour [13], with turtles digging and trampling sand in periodic bursts that accumulate lactic acid, followed by resting until the lactate lowers or until stimulated by activity from the bottom turtles [3,11,14,15].

However, our understanding of movement behaviours, periodic digging and diel activity patterns during the hatching-to-emergence period has been constrained by the challenges of observing a natural life stage that occurs in a concealed environment. The introduction of artificial light, daylight cues and viewing panes that hatchlings would typically not have in a natural nest might influence their digging behaviour. For example, being pressed up against a hard, smooth surface rather than completely surrounded by sand may favour alternate limb crawling behaviour while digging or may lead to the formation of air pockets that could alter digging behaviour. Similarly, introduction of light and daylight during observation could create a ‘circadian cue’ for digging hatchlings that does not exist deep in a natural nest. As hatchlings in a natural nest approach the surface (approximately 15 cm), it is thought that thermal variations provide cues for the cessation of activity in order to avoid emerging during the day [16,17]. Indeed, recent work with acoustic microphones suggests that diel patterns in digging do not occur deep (approximately 70 cm) in the nest 1–2 days post-hatching where temperatures are uniform but become more apparent as they near the surface (approximately 40 cm), and temperature fluctuations become pronounced [1,18]. In addition to the challenges of observing hatchlings during emergence, it is difficult to predict the precise timing to begin observations due to the somewhat unpredictable nature of the timing of hatching [19,20]. Unfortunately, our lack of information about the hatching-to-emergence phase prevents understanding how nesting conditions and hatchling traits impact hatchling behaviour and survival, thereby obscuring the consequences of maternal nesting behaviour and conservation-driven nest interventions [21].

The advent of miniaturized wildlife accelerometry provides a novel opportunity to observe the intricacies of post-hatch activity within underground nests. Accelerometers placed directly on animals has exploded as a research methodology, allowing researchers to describe and detect behaviours, as well as quantify energy expenditure, without having to make direct observations [22–26]. For example, devices affixed to wild spotted hyenas (*Crocuta crocuta*) have been used to ascertain activity patterns, such as energy compensation by tracking periods of high activity followed by low-activity rest days, behavioural variation among individuals and social synchronization of behaviours [27]. Similarly, accelerometers placed on nesting green turtles (*Chelonia mydas*) were able to detect behaviours such as egg laying and estimate the number of eggs laid by identifying back and forth movements [28]. Moreover, in several species (e.g. Great cormorants, Magellanic penguins and Larger hairy armadillos) accelerometers have been used to quantify energy expenditure by coupling acceleration data with oxygen consumption [29–32]. Thus, accelerometers placed on hatchlings could allow recording temporal patterns in movement, as well as describing movement behaviours. In addition, using accelerometers to estimate energy expenditure in digging hatchlings would allow examining how the timing and location of nests impact energetic reserves in emergent hatchlings. For example, one could test the hypothesis that clutch size reduces individual energetic cost of digging out, thereby enhancing hatchling fitness [12,13,15,33]. The technology offers numerous palpable benefits, including cost-effective and non-invasive monitoring of animals, robust quantitative data of movement and behaviours, and crucially, the ability to study animals in scenarios where their visibility is limited or direct observation may disrupt their natural behaviour [34].

In this study, we aim to provide proof-of-concept for using an innovative technological approach to observe the cryptic period of hatching-to-emergence within green turtle nests. Our new approach employs a hatching detector [35] to enable affixing a tri-axial accelerometer directly to the carapace of newly hatched green turtles and subsequently recording their movement during natural nest emergence. The accelerometers allow, for the first time, observing hatchling digging behaviour in a natural nest. We use the accelerometers to reveal the movements and temporal activity patterns in hatchling green turtles in this environment.

## 2. Methods

### (a) Study site

This study was conducted during the 2022–2023 nesting season on Heron Island, Queensland, Australia (S 23.44307°, E 151.91809°). Heron Island is a long-term monitoring nesting site for green turtles in the southern Great Barrier Reef, which nest on the island from November to April [36]. We identified study nests on the east-facing side of the island at Shark Bay between 7 and 16 December 2022. As part of a larger study on the effects of nest relocation and shading, several of this study’s nests (*n* = 7/10) were relocated immediately post-oviposition to a site within Shark Bay but approximately 50 metres inland from the low tide line and surrounded by a barrier fence that allowed hatchlings to escape but prevented nesting turtles from entering. The site was within the range of green turtles’ natural nesting. Relocated nests were 60 cm deep. Each relocated nest received a HOBO MX2307 Bluetooth temperature probe to allow predicting the approximate hatching time from downloaded nest temperature traces [35]. The depth of natural nests was not measured due to the presence of the nesting female and the difficulty of predicting where the sand surface would be upon nest completion. In addition, subsequent nesting females often move sand around and alter the depth of nests after construction. Similarly, temperature probes could not be installed in natural nests because the wires and dataloggers on the surface could have been disturbed by subsequent nesting females.

### (b) Hatching detectors and accelerometers

**Figure 1.**
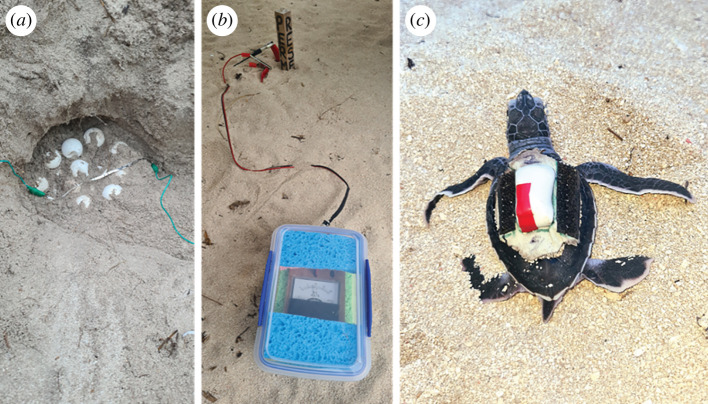
(*a*) Hatching detector consisting of a 150 × 2 mm strip of aluminium foil placed at the top of the egg clutch and secured with an alligator clip on each side. (*b*) Voltmeter used to measure voltage of the circuit at the surface. (*c*) Green turtle hatchling with accelerometer affixed to the carapace.

We recorded hatchling movement immediately after hatching until emergence at the sand surface for 10 nests in February 2023. To do this, we first estimated a time window of hatching (using the HOBO MX2307 nest temperature data [35]) and excavated the nests by hand a few days prior, keeping track of sand layers, which typically differ in moisture. We deployed ‘hatching detectors’ [35] into the nests (including the three non-relocated nests without HOBO probes) as follows. Upon excavation, the top eggs in each nest were confirmed to be unhatched. A 150 × 2 mm strip of aluminium foil was placed across the top eggs within a clutch, and two alligator clips on each side of the strip were connected to the wires leading to the surface (figure 1*a*). The nest was backfilled by first replacing the deep moist sand and then following with the originally higher layers of sand. Nests were checked for hatching 6–8 times a day at roughly 3 h intervals by connecting a voltmeter in tandem with a 9 volt transistor battery and 1000 ohm resistor to create a series circuit (figure 1*b*). Hatchlings emerging from the top eggs cause the foil strip to break, leading to a drop in voltage when checking the circuit at the sand surface.

Upon detection of hatching, the nest was excavated until the first hatchling was found on the top of the clutch within the nest chamber. For each nest (*n* = 10), an Axy 5 XS bio-logger (21.5 × 11.5 × 7.00 mm; 2 g; Technosmart Europe srl, https://www.technosmart.eu/) was affixed to one hatchling with VetBond glue (figure 1*c*). The clutch, including the monitored hatchling, were subsequently reburied using the same process as above. The accelerometer was calibrated to measure raw tri-axial acceleration (*g*) between −2 and 2 *g*. We used a sample rate of 1 Hz, which is regarded as a practicable frequency for small animals [37]. The accelerometer was recovered by capturing emergent hatchlings using a corral deployed on the sand surface around the nest cavity and checking the corral every 3 h. To compare accelerometer signal output between in-nest activity (i.e. digging) and surface crawling, one emergent hatchling that carried an accelerometer was allowed to crawl on a flat sand surface for 60 s.

**Figure 2.**
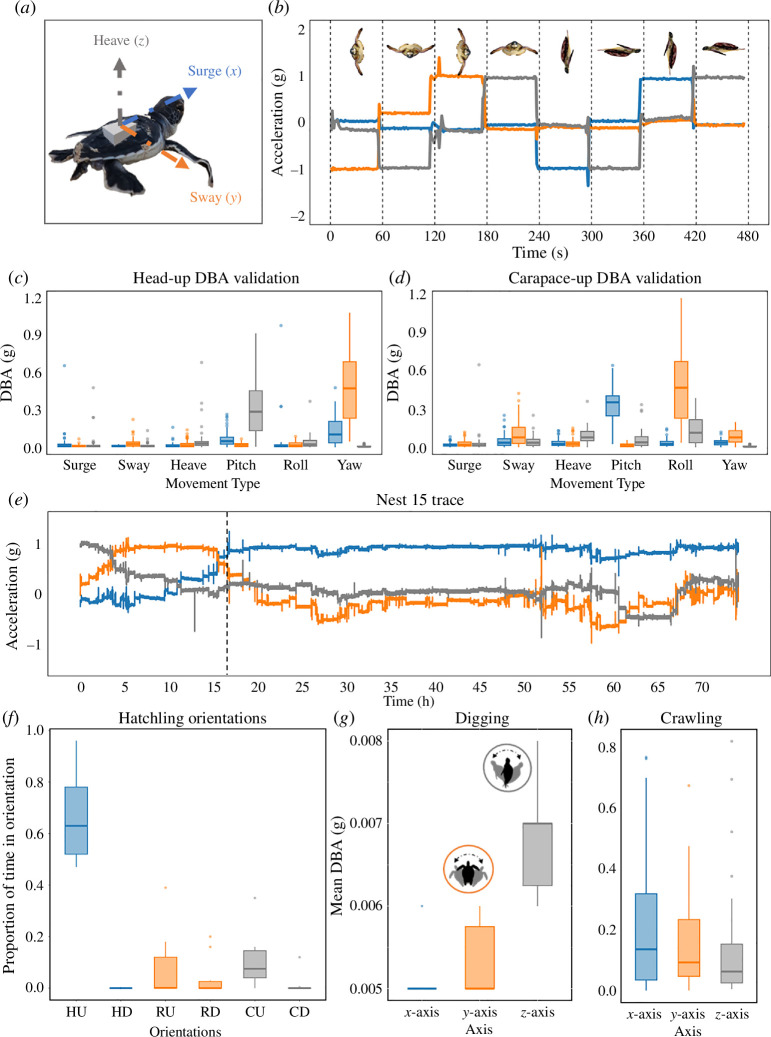
(*a*) Diagram of a green turtle with an accelerometer affixed to its carapace depicting each accelerometry axis. (*b*) Validation trace of an accelerometer in each orientation. (*c*) and (*d*) Dynamic body acceleration (DBA) for different movement types in head-up and carapace-up orientations. (*e*) Raw accelerometer trace of Nest 15. The vertical black line indicates the time that the hatchling orientates in a head-up position. (*f*) Proportion of time that digging hatchlings spent in each body orientation: (HU = Head up; HD = Head down; RU = Right-side up; RD = Right-side down; CU = Carapace up; CD = Carapace down). (*g*) Dynamic body acceleration (movement) of 10 hatchlings for each axis of acceleration whilst digging out of the nest. (*h*) Dynamic body acceleration (movement) of 1 hatchling whilst crawling.

Body orientation with respect to the Earth’s gravitational field (i.e. 1 *g* is Earth’s gravitational pull) was revealed by raw acceleration values in three axes, surge (*x*-axis), sway (*y*-axis) and heave (*z*-axis; figure 2*a*). Based on the position of the accelerometer fixed to the hatchling’s carapace, we can distinguish six primary orientations: head-up (*x* = 1 *g*); head-down (*x* = −1 *g*); carapace-up (*z* = 1 *g*); carapace-down (*z* = −1 *g*); and right-side-up (*y* = 1 *g*); right-side-down (*y* = −1 *g*) (see figure 2*b* for illustration of these orientations). We validated these orientations for our accelerometer placement on the hatchling by rotating the hatchling through each axis slowly over a minute. We stopped rotating the hatchling at every 90° interval and continued until a 360° rotation of the device was completed (figure 2*b*).

Movement activity and intensity can be estimated using dynamic body acceleration (DBA; see calculations for DBA and VeDBA below). We validated the dynamic movement components in the three axes by affixing an accelerometer to a book and moving it in surge, sway and heave movements for 60 s at a time while on a smooth surface and adjacent to a wall to reduce potential noise. Additionally, we validated angular acceleration by moving the accelerometer in pitch, roll and yaw movements with the head of a hatchling imitating the ‘prow’ of a ship. We performed these six movement types in two different body orientations: ‘head-up’ and ‘carapace-up’ (see figure 2). Our movements of the accelerometer were imperfect in their isolation of the six movement types and were not standardized in intensity, but insight can be gained by comparing DBA across axes. Importantly, DBA is relatively insensitive to movement around any axis with raw acceleration values near 1 or −1 [38]. When the hatchling is in a ‘head-up’ position (*x* = 1), the accelerometer is not very sensitive to movement in the *x*-axis, while the *y*-axis records sway and yaw (tipping body left or right), and the *z*-axis records heave and pitch (tipping body dorsoventrally; figure 2*c*). Roll (spinning like a top) is not recorded since it is rotation around the vertical axis. In contrast, in a ‘carapace-up’ orientation, the *x*-axis records changes in pitch (tipping body dorsoventrally), the *y*-axis records roll (tipping onto right or left side) and yaw is not recorded as it is rotation around the vertical axis (figure 2*d*).

### (c) Analysis

R v. 4.3.1 and MATLAB R2023a v. 9 were used to process accelerometry data and conduct statistical analyses. First, the raw accelerometer data from each hatchling were cleaned to remove values that were recorded when transporting the accelerometer or handling the hatchling. To characterize the most common body orientations within the nest, we summarized the raw accelerometer data by filtering values on each axis (*x*, *y*, *z*) to include only those that exceeded a threshold (≥ |0.70|*g*) in order to focus on clearly delimited categories of orientation. Records at lower absolute values represent intermediate orientations that are difficult to interpret. We calculated the total proportion of all records in each of the six hatchling orientations described in (figure 2*b*).

To quantify movement activity and intensity in the three axes, we first calculated DBA in each axis. We used the R package *zoo* [39] to apply a running mean over 3 s across each axis (also known as the static acceleration (SA); [31]). DBA was then calculated for each second by subtracting SA from the raw accelerometer record:


$$\begin{array}{l} DBA=(Raw\;Data\;-SA) \end{array}$$

For each trial, we averaged the DBA within each axis to compare the intensity of movement among axes during the hatching-to-emergence period.

Subsequently, we calculated the total intensity of movement across all axes using the vectorial dynamic body acceleration (VeDBA) [40]:


$$VeDBA=\sqrt{\left(X_{DBA}^{2}+Y_{DBA}^{2}+Z_{DBA}^{2}\right)}$$

**Figure 3.**
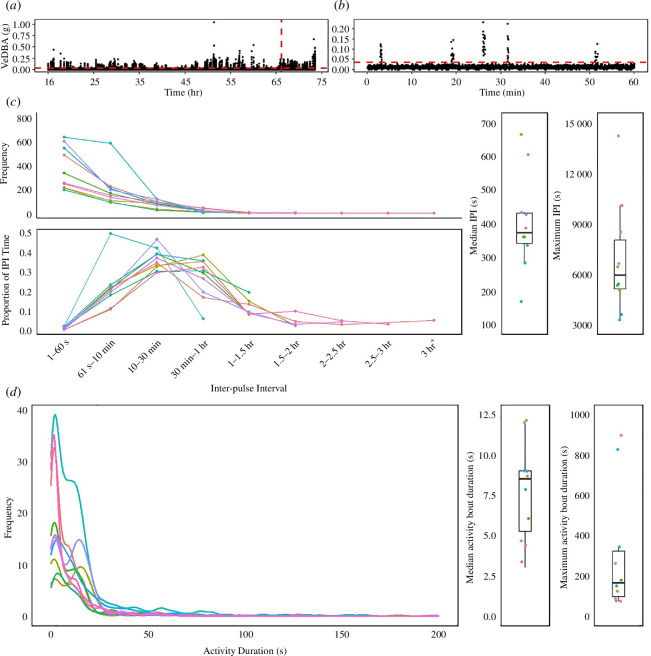
(*a*) Scatterplot of vectorial dynamic body acceleration (VeDBA) over time for Nest 15 beginning from when the hatchling oriented itself head-up. The vertical red line indicates the start time of the time scale presented in (*b*). The horizontal red dashed line indicates the noise threshold filter (0.035). (*b*) An enhanced image of plot (*a*) from 66 to 67 h, truncated for VeDBA values < 0.25. (*c*) Frequency distribution of IPIs in each of the 10 nests and proportion of IPI time spent binned into IPI categories from 1 s–3 h+ with median and max IPI values for each of the 10 nests. (*d*) Frequency distribution, median and maximum of activity bout durations that were filtered with a 1 min IPI threshold (frequency distribution truncated at 200 s).

Motivated by previous observations that digging involves discrete periods of active digging separated by periods of rest [11], we used VeDBA to identify bouts of movement activity and inactivity in order to quantify the duration of inactivity. First, we excluded all data prior to the time when the hatchling first reached our threshold of ‘head-up’ (*x* ≥ 0.7 *g* for at least 10 consecutive s; see results). We excluded the initial body reorientation phase (see results) because the movement during this time does not strictly represent digging. Second, we filtered out background noise in VeDBA. To establish the background noise in our accelerometer data, we buried accelerometers at the relocation site at three different depths (20, 40 and 60 cm) for 1 h with the same 1 Hz sampling rate as in our nest deployments. Background noise in this environment was VeDBA < 0.02. We also visually inspected the VeDBA scatterplots and VeDBA frequency distributions of each nest to assess apparent noise. We chose a noise filter of VeDBA = 0.035 because this removed the obvious noise (see, for example, figure 3*a*,*b*). ‘Pulses’ in movement activity were defined as VeDBA values above the noise threshold. ‘Inter-pulse intervals (IPI)’ were defined as any time step greater than 1 s between pulses. To quantify the distribution of IPIs within the nest escape period, we binned the IPIs of each nest into nine intuitive time intervals (1–60 s; 61 s–10 min; 10–30 min; 30 min–1 h; 1–1.5 h; 1.5–2 h; 2–2.5 h; 2.5–3 h; 3 h+). We calculated the number of IPIs and the proportion of total time spent in each of these nine intervals for each nest (figure 3*d*). Our conclusions regarding the distribution of IPIs were not altered substantially with alternative noise filters of VeDBA = 0.045, 0.055; however, a noise filter of VeDBA = 0.025 appeared to be too low (electronic supplementary material).

To quantify durations of activity bouts, we searched for an operational definition of the minimum IPI to delineate the end of an activity bout and the start of an inactive (i.e. rest) period. Given the lack of prior knowledge on digging and resting behaviour, we visually inspected the frequency distributions of IPIs to search for duration of IPIs that could provide this operational definition (i.e. small IPIs indicate the same activity bout, while large IPIs indicate a defined rest period). However, the IPI data provided no clear guidance for an operational definition (see results). Instead, we chose a range of IPIs to delineate activity bouts (1, 5 and 10 min) and report the activity bout durations for each of these thresholds.

To determine whether there were diel patterns of activity, we averaged VeDBA data within six 4 h time blocks for each nest (12.00–16.00; 16.00–20.00; 20.00–24.00; 00.00–04.00; 04.00–08.00; 08.00–12.00) and for the first and last 24 h for each nest. We removed two nests from the analyses due to data overlap in the first and last 24 h period. Even though the data were repeated across time blocks for each nest, we treated the data points from each nest as independent for statistical analysis owing to the small number of nests in our sample (*n* = 8). We then performed a two-factor ANOVA (*lm* function) to test for differences in average VeDBA among time blocks and 24 h periods, including an interaction between time block and period. Because the interaction was significant (see results), we ran separate ANOVAs for each period (first and last 24 h) examining whether average VeDBA varied among time blocks.

## 3. Results

### (a) Overall emergence patterns

The time between attaching the accelerometer to a hatchling and the emergence of this hatchling ranged from 31 to 92 h (median (natural, *n* = 3) = 76.2 ± 1.0 h; median (relocated, *n* = 7) = 73.9 ± 21.0 h). The first hatchling emergence (including the accelerometer hatchling) occurred during night-time hours (19.00–04.00) for all nests. At this first emergence, which would have occurred during a maximum 3 h time window, a large proportion of the clutch was present (64–94% of live hatchlings; median (natural) = 90%, median (relocated) = 80%). During the 48 h post-emergence nest excavation, few live hatchlings were found remaining in the sand column or in the nest cavity (2–15; median (natural) = 2, median (relocated) = 7), indicating most hatchlings had emerged within 48 h of first emergence.

### (b) Orientation and axes of movement

During the entire recording phase, the 10 hatchlings spent the greatest proportion of time in the head-up orientation (67%), with much less time spent in head-down (0%), right-side-up (8%), right-side-down (4%), carapace-up (10%) and carapace-down (1%) orientations (figure 2*f*; values do not add up to 100% because some orientations were intermediate). In the initial phases of each trace, the hatchling had a carapace-up (*z* ≥ 0.7 *g*) orientation that matched how we placed them at the top of the nest chamber after affixing the accelerometer. Subsequently, every turtle went through a transition in orientations (e.g. sometimes on their sides) before acquiring and maintaining the ‘head-up’ orientation (see figure 2*e* for an example accelerometry trace). This initial phase lasted 9.1 ± 8.7 h (range 0–28 hr), leaving 67.9 ± 20.6 h of head-up time within the nest to analyse.

After acquiring the ‘head-up’ orientation, movement activity based on DBA primarily occurred within the *z*-axis, which represents pitching dorsoventrally (figure 2*g*). A smaller amount of movement was recorded in the *y*-axis, which represents yaw (tipping left to right). DBA in the *x*-axis was small, which is to be expected with a head-up orientation. In contrast, crawling on the sand surface involved similar levels of left-right movement (*y*-axis rolling) and dorsoventral pitching (recorded in the *x*-axis in the carapace-up orientation; figure 2*h*).

### (c) Activity and inactive periods (inter-pulse interval)

When looking at a compressed time scale, i.e. over the entire hatch to surface emergence period, hatchlings appeared to be active nearly continuously as quantified by VeDBA (figure 3*a*); however, when looking at an expanded time scale, it is apparent that there are short active movements separated by variable IPIs (Figure 3*b*). The duration of IPIs were skewed, with a median of approximately 7 min, but maximum IPIs lasted 54–236 min (figure 3*c*). The majority of IPIs were less than 1 min long; however, individuals had most of their inactivity durations within the 10 min–1 h classification (figure 3*c*). The activity bouts were generally short in duration, with medians of 8.5 s (maximum, 1.5–15 min; 1 min IPI threshold), 12 s (maximum, 11–55 min; 5 min IPI threshold), 3 min (maximum, 25 min–2 h; 10 min IPI threshold) (figure 3*d*).

### (d) Diel patterns in activity

**Figure 4.**
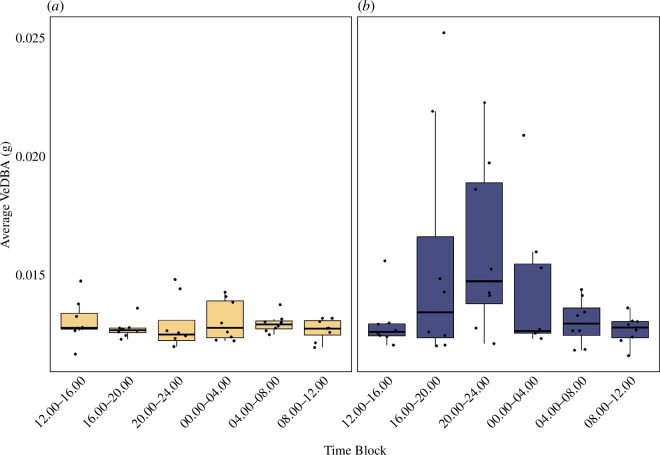
Average vectorial dynamic body acceleration (VeDBA) pooled into six 4 h time blocks for each nest (*n* = 8). (*a*) First 24 h period after acquiring a head-up orientation. (*b*) Last 24 h period prior to emergence.

When the first and last 24 h of activity were considered together, we found a near significant interaction between 24 h period and diel time block (interaction, *F* = 2.07 ; *p* = 0.074), as well as significant main effects of 24 h period (*F* = 8.28; *p* = 0.0052) and near significant effects of time block (time block, *F* = 1.95; *p* = 0.09; figure 4) on average VeDBA. When looking at the first and last 24 h periods separately, we found that there was no significant difference in activity across time blocks in the first 24 h after hatching (*F* = 0.37, *p* = 0.86; Figure 4), but there was a near significant difference in activity for time blocks in the 24 h before emergence onto the sand surface (*F* = 2.12, *p* = 0.08; Figure 4).

## 4. Discussion

The cryptic-yet-crucial life stage of sea turtle hatchlings concealed beneath a metre of sand is a challenge to study. However, using a minimally invasive approach (hatching detectors and accelerometers), we were able to observe hatchling sea turtle movement behaviour and intensity during the multi-day period of nest escape. With this approach, we uncovered several surprising findings in hatchling orientation, movement, and activity timing that fundamentally change and add to our understanding of the hatching-to-emergence life stage. Unexpectedly, hatchlings in our study required only 1.5–4 days to emerge from the nest (including the natural nests), which is substantially shorter than the previous estimates of 3–7 days [1,2]. Possibly, our use of a hatching detector [35] allowed more precise recording of hatching time. However, it is worth noting that nest excavation may have subsequently changed the characteristics of the sand column by loosening the sand, making it easier to dig out.

We discovered a level of control in orientation and movement that does not match the previous description of generalized ‘thrashing’ [12]. In particular, hatchlings consistently maintained a head-up orientation and moved primarily in the heave axis (pitching dorsoventrally). Significantly, maintaining a head-up orientation ensures that hatchling movement leads to ascent through the sand column towards the surface. The consistency in head-up orientation suggests new lines of inquiry regarding orientation mechanisms while underground. In particular, orientation behaviour is likely guided by the Earth’s gravitational force (negative geotaxis), considering the general absence of other cues. Sea turtles are known to use the Earth’s gravitational force for orientation post-emergence when moving downhill even in the absence of primary cues such as light and silhouettes [41] and our within-nest observations now suggest that they can also use gravitational force as a body orientation cue. For subterranean animals, sensory stimuli other than light are often used for navigation. For example, the blind mole rat (*Spalax ehrenbergi*) utilizes magnetoreception as a primary underground navigational cue [42], and Oriental hornets (*Vespa orientalis*) construct their combs using negative geotaxis in the absence of light [43]. Further examination of geotaxis would be useful in advancing our understanding of hatchling orientation cues within the nest.

Our observation that movement was mostly dorsoventral pitching is contrary to common terrestrial crawling movement patterns. The typical Cheloniid pattern of terrestrial locomotion involves diagonally opposite limbs protracting and retracting [44], which our hatchling-born accelerometer recorded as equal amounts of left-to-right rolling movement as dorsoventral pitching. Alternating limb movement has also been recorded in head-up bipedal animals (e.g. penguins) and is associated with tipping left-to-right (i.e. yaw) [38]. In contrast, head-up digging hatchlings in our study displayed very little left-to-right (yaw) movement, instead using a pitching movement one might expect from simultaneous limb movement. In aquatic environments, Cheloniid hatchlings simultaneously protract their forelimbs to propel themselves in water, i.e. they ‘fly’ through the water [45,46]. Thus, based on our data, it appears that hatchlings dig in a bilateral, pitching motion, effectively ‘swimming’ vertically to the sand surface. However, direct comparison of movement between within-nest digging and surface crawling hatchlings is challenging owing to differences in orientation, which changes the sensitivity and interpretation of movement in each axis ([38] and herein). A more suitable comparison could be drawn from a turtle crawling up a near vertical gradient or swimming vertically. The differences between our findings on hatchling body movement and previous observations may be due to the lack of a viewing pane [11], which may promote different modes of digging. A hard vertical surface (i.e. viewing pane) may prevent heaving and make swaying left and right more effective. In addition, if glass window observation experiments used sand with high moisture content, the sand would compact, requiring scratching to loosen it. In contrast, drier sand easily collapses and flows like a liquid, which may favour ‘swimming’ through the sand, aligning with our accelerometry observations.

Surprisingly, hatchling movement activity displayed less periodicity and more continuity than expected, with IPIs exhibiting a continuous distribution skewed towards very short intervals (<60 s; see also [47]). However, despite the high frequency of short intervals, the greatest proportion of time spent inactive was within intervals of 10 min–1 h. Based on anecdotal observations [12], we expected regular, extended IPIs that allow clearing blood lactate that accumulates during high-intensity anaerobic activity to replenish their oxygen debt. This temporal pattern of digging is supported by previous observations of high blood lactate levels in hatchlings actively digging out of the nest, yet low blood lactate found in hatchlings at rest in the egg chamber or just below the surface [3]. In contrast, periodic spikes in oxygen consumption have been observed in laboratory experiments of digging hatchlings [13], indicating aerobic metabolism is employed to some extent. Furthermore, both aerobic and anaerobic metabolism are utilized by hatchlings during their crawl to the ocean and the frenzied swim in the sea [48]. Thus, the high variability in IPIs observed in our study indicate further investigation is needed on hatchling movement intensity with respect to the use of aerobic versus anaerobic metabolism.

Our data provide new insight into the assumption that hatchlings in the sand column dig primarily at night. An increase in night-time activity in sea turtle hatchlings has been reported from visual observations where daylight cues were introduced to the nest [11] but also using microphones buried within the nest [18]. Here, we found greater night-time activity only during the last 24 h before nest emergence when hatchlings were closer to the sand surface and may have gained more information on diel cues through thermal or light variation. Activity during the first 24 h showed no diel patterns. The assumption that hatchlings have a sense of diel time when near the sand surface is also supported by wider observations that sea turtle hatchlings emerge primarily during the night [16,49]. Our study found similar results in agreement with previous research on Heron Island (Gyuris [16]). However, in natural nests, diel movement activity patterns near the surface may be driven by temperature in addition to (or rather than) light [16]. High sand temperatures can inhibit coordinated muscle movements of green turtle hatchlings and inhibit the emergence process [50]. Indeed, emergence has been recorded distributed throughout 24 h when rainy and overcast weather keep sand surface temperatures below 30°C [16].

Our findings on hatchling green turtles provide a new understanding of the cryptic hatching-to-emergence life stage of sea turtles. Accordingly, our study demonstrates the substantial opportunity accelerometers represent to investigate additional aspects of this stage in sea turtles. For example, accelerometers could be used to examine how variation in sand water content and thermal gradients within the nest influences the nest escape process. Combining accelerometry data while digging with concurrent measurement of oxygen consumption [13] would allow validating the relationship between accelerometer output (VeDBA) and energy expenditure. With this validation, one could estimate the energetic cost of nest escape in natural nests and test hypotheses regarding, for example, social facilitation of digging, optimal maternal nest site selection and benefits of sand moisture. Moreover, the hatching-to-emergence phase of a sea turtle’s life is just one example where a critical life stage occurs in a cryptic environment. Observing animals in an obscured subterranean environment is a challenge across many species [51]. Using accelerometers to describe behaviours such as digging and to estimate energetic expenditure during life stages that are cryptic could reveal crucial links between phenotypic variation and habitat characteristics such as sand moisture and nest depth [22].

Incubation conditions have a profound influence on the phenotype of young reptiles [7,52,53], yet nest conditions may further impact emergent hatchlings during the digging-out phase in unknown ways. Successful emergence and energetic expenditure could be influenced by many factors, including nest depth, temperature, moisture levels, sand texture, grain size and the extent of root penetrance [54,55]. Understanding the importance of these characteristics for hatchlings in the hatching-to-emergence phase would inform research into nesting ecology as well as conservation mitigation strategies at the nest stage. In particular, nest relocation has been shown to be a simple and effective tool in mitigating several key threats to hatchling sea turtles. However, manipulation of the nest characteristics such as substrate and depth could have underlying consequences for hatchlings that we currently do not understand. To generalize our findings, further investigations are warranted at diverse nesting sites, sand water content and with various sea turtle species.

## Data Availability

The supporting dataset for this research has been deposited in Dryad Digital Repository [56]. Supplementary material is available online [57].

## References

[1] Balazs GH , Ross E . 1974 Observations on the preemergence behavior of the green turtle. Copeia **1974** , 986. (10.2307/1442606)

[2] Hamann M , Jessop TS , Schäuble CS . 2007 Fuel use and corticosterone dynamics in hatchling green sea turtles (Chelonia mydas) during natal dispersal. J. Exp. Mar. Biol. Ecol. **353** , 13–21. (10.1016/j.jembe.2007.08.017)

[3] Baldwin J , Gyuris E , Mortimer K , Patak A . 1989 Anaerobic metabolism during dispersal of green and loggerhead turtle hatchlings. Comp. Biochem. Physiol. A Physiol. **94** , 663–665. (10.1016/0300-9629(89)90613-0)

[4] Adams DM , Williamson SA , Evans RG , Reina RD . 2022 Increasing hypoxia progressively slows early embryonic development in an oviparous reptile, the green turtle, Chelonia mydas. R. Soc. Open Sci. **9** , 220709. (10.1098/rsos.220709) 36061518 PMC9428527

[5] Booth DT . 2017 Influence of incubation temperature on sea turtle hatchling quality. Integr. Zool. **12** , 352–360. (10.1111/1749-4877.12255) 28054446

[6] Bladow RA , Milton SL . 2019 Embryonic mortality in green (Chelonia mydas) and loggerhead (Caretta caretta) sea turtle nests increases with cumulative exposure to elevated temperatures. J. Exp. Mar. Biol. Ecol. **518** , 151180. (10.1016/j.jembe.2019.151180)

[7] Noble DWA , Stenhouse V , Schwanz LE . 2018 Developmental temperatures and phenotypic plasticity in reptiles: a systematic review and meta-analysis. Biol. Rev. **93** , 72–97. (10.1111/brv.12333) 28464349

[8] São Miguel RAM , Anastácio R , Pereira MJ . 2022 Sea turtle nesting: what is known and what are the challenges under a changing climate scenario. Open J. Ecol. **12** , 1–35. (10.4236/oje.2022.121001)

[9] Putman NF , Naro-Maciel E . 2013 Finding the ‘lost years’ in green turtles: insights from ocean circulation models and genetic analysis. Proc. R. Soc. B **280** , 20131468. (10.1098/rspb.2013.1468) PMC3757977 23945687

[10] Gibbons JW . 2013 A long-term perspective of delayed emergence (aka overwintering) in hatchling turtles: some they do and some they don’t, and some you just can’t tell. J. Herpetol. **47** , 203–214. (10.1670/12-122)

[11] Carr AF , Ogren LH . 1960 The ecology and migrations of sea turtles. 4, The green turtle in the Caribbean Sea. Bull. AMNH **121** , 22–23.

[12] Carr A , Hirth H . 1961 Social facilitation in green turtle siblings. Anim. Behav. **9** , 68–70. (10.1016/0003-3472(61)90051-3)

[13] Rusli MU , Booth DT , Joseph J . 2016 Synchronous activity lowers the energetic cost of nest escape for sea turtle hatchlings. J. Exp. Biol. **219** , 1505–1513. (10.1242/jeb.134742) 27207954

[14] Hendrickson JR . 1958 The green sea turtle, Chelonia mydas (Linn.) in Malaya and Sarawak. In Proc. of the Zoological Society of London, vol. **130** , pp. 455–535, Oxford, UK: Blackwell Publishing Ltd.

[15] Carr AF , Ogren LH . 1959 The ecology and migrations of sea turtles. 3, Dermochelys in Costa Rica. Am. Mus. Nov. 14–16.

[16] Gyuris E . 1993 Factors that control the emergence of green turtle hatchlings from the nest. Wildl. Res. **20** , 345–353. (10.1071/WR9930345)

[17] Salmon M , Reising M . 2014 Emergence rhythms of hatchling marine turtles: is a time sense involved? Chelon. Conserv. Biol. **13** , 282–285. (10.2744/CCB-1121.1)

[18] Nishizawa H , Hashimoto Y , Rusli MU , Ichikawa K , Joseph J . 2021 Sensing underground activity: diel digging activity pattern during nest escape by sea turtle hatchlings. Anim. Behav. **177** , 1–8. (10.1016/j.anbehav.2021.04.013)

[19] Clabough EBD , Kaplan E , Hermeyer D , Zimmerman T , Chamberlin J , Wantman S . 2022 The secret life of baby turtles: a novel system to predict hatchling emergence, detect infertile nests, and remotely monitor sea turtle nest events. PLoS One **17** , e0275088. (10.1371/journal.pone.0275088) 36288397 PMC9605334

[20] Hinsley C , Delaney DM , Janzen FJ . 2021 Use of accelerometers to monitor subterranean movement is challenging for small reptile nests. Herpetol. Rev. **51** , 702–707.

[21] van de Merwe JP , Ibrahim K , Whittier JM . 2013 Post‐emergence handling of green turtle hatchlings: improving hatchery management worldwide. Anim. Conserv. **16** , 316–323. (10.1111/j.1469-1795.2012.00603.x)

[22] Brown DD , Kays R , Wikelski M , Wilson R , Klimley AP . 2013 Observing the unwatchable through acceleration logging of animal behavior. Anim. Biotelem. **1** , 1–16. (10.1186/2050-3385-1-20)

[23] Leos‐Barajas V , Photopoulou T , Langrock R , Patterson TA , Watanabe YY , Murgatroyd M , Papastamatiou YP . 2017 Analysis of animal accelerometer data using hidden Markov models. Methods Ecol. Evol. **8** , 161–173. (10.1111/2041-210X.12657)

[24] Hounslow JL , Fossette S , Byrnes EE , Whiting SD , Lambourne RN , Armstrong NJ , Tucker AD , Richardson AR , Gleiss AC . 2022 Multivariate analysis of biologging data reveals the environmental determinants of diving behaviour in a marine reptile. R. Soc. Open Sci. **9** , 211860. (10.1098/rsos.211860) 35958091 PMC9364005

[25] Lennox RJ , Eldøy SH , Dahlmo LS , Matley JK , Vollset KW . 2023 Acoustic accelerometer transmitters and their growing relevance to aquatic science. Mov. Ecol. **11** , 45. (10.1186/s40462-023-00403-3) 37501158 PMC10375738

[26] Morgan A , Christensen C , Bracken AM , O’Riain MJ , King AJ , Fürtbauer I . 2023 Effects of accelerometry-derived physical activity energy expenditure on urinary C-peptide levels in a wild primate (Papio ursinus). Horm. Behav. **152** , 105355. (10.1016/j.yhbeh.2023.105355) 37031555

[27] Minasandra P , Jensen FH , Gersick AS , Holekamp KE , Strauss ED , Strandburg-Peshkin A . 2023 Accelerometer-based predictions of behaviour elucidate factors affecting the daily activity patterns of spotted hyenas. R. Soc. Open Sci. **10** , 230750. (10.1098/rsos.230750) 38026018 PMC10645113

[28] Jeantet L , Hadetskyi V , Vigon V , Korysko F , Paranthoen N , Chevallier D . 2022 Estimation of the maternal investment of sea turtles by automatic identification of nesting behavior and number of eggs laid from a tri-axial accelerometer. Animals **12** , 520. (10.3390/ani12040520) 35203228 PMC8868198

[29] Enstipp MR , Ciccione S , Gineste B , Milbergue M , Ballorain K , Ropert-Coudert Y , Kato A , Plot V , Georges JY . 2011 Energy expenditure of freely swimming adult green turtles (Chelonia mydas) and its link with body acceleration. J. Exp. Biol. **214** , 4010–4020. (10.1242/jeb.062943) 22071193

[30] Halsey LG , Shepard ELC , Quintana F , Gomez Laich A , Green JA , Wilson RP . 2009 The relationship between oxygen consumption and body acceleration in a range of species. Comp. Biochem. Physiol. A Mol. Integr. Physiol. **152** , 197–202. (10.1016/j.cbpa.2008.09.021) 18854225

[31] Halsey LG , Shepard ELC , Wilson RP . 2011 Assessing the development and application of the accelerometry technique for estimating energy expenditure. Comp. Biochem. Physiol. A Mol. Integr. Physiol. **158** , 305–314. (10.1016/j.cbpa.2010.09.002) 20837157

[32] Halsey LG , Jones TT , Jones DR , Liebsch N , Booth DT . 2011 Measuring energy expenditure in sub-adult and hatchling sea turtles via accelerometry. PLoS One **6** , e22311. (10.1371/journal.pone.0022311) 21829613 PMC3150346

[33] Rusli MU , Booth DT . 2016 Bigger clutch sizes save offspring energy during nest escapes. Behav. Ecol. Sociobiol. **70** , 607–616. (10.1007/s00265-016-2079-1)

[34] Smith JE , Pinter-Wollman N . 2021 Observing the unwatchable: integrating automated sensing, naturalistic observations and animal social network analysis in the age of big data. J. Anim. Ecol. **90** , 62–75. (10.1111/1365-2656.13362) 33020914

[35] Booth DT , Turner AG , Laloë JO , Limpus CJ . 2022 How well do embryo development rate models derived from laboratory data predict embryo development in sea turtle nests? J. Exp. Zool. A Ecol. Integr. Physiol. **337** , 516–526. (10.1002/jez.2585) 35189044 PMC9305169

[36] Chaloupka M , Limpus C . 2001 Trends in the abundance of sea turtles resident in southern Great Barrier Reef waters. Biol. Conserv. **102** , 235–249. (10.1016/S0006-3207(01)00106-9)

[37] Gleiss AC , Wilson RP , Shepard ELC . 2011 Making overall dynamic body acceleration work: on the theory of acceleration as a proxy for energy expenditure. Methods Ecol. Evol. **2** , 23–33. (10.1111/j.2041-210X.2010.00057.x)

[38] Shepard E *et al* . 2010 Identification of animal movement patterns using tri-axial accelerometry. Endang. Species Res. **10** , 47–60. (10.3354/esr00084)

[39] Grothendieck G , Zeileis A . 2005 Zoo: s3 infrastructure for regular and irregular time series. J. Stat. Softw. **14** , 27. (10.18637/jss.v014.i06)

[40] Qasem L , Cardew A , Wilson A , Griffiths I , Halsey LG , Shepard ELC , Gleiss AC , Wilson R . 2012 Tri-axial dynamic acceleration as a proxy for animal energy expenditure; should we be summing values or calculating the vector? PLoS One **7** , e31187. (10.1371/journal.pone.0031187) 22363576 PMC3281952

[41] Salmon M , Wyneken J , Fritz E , Lucas M . 1992 Sea finding by hatchling sea turtles: role of brightness, silhouette and beach slope as orientation cues. Behaviour **122** , 56–77. (10.1163/156853992X00309)

[42] Kimchi T , Terkel J . 2001 Magnetic compass orientation in the blind mole rat ‘Spalax ehrenbergi’. J. Exp. Biol. **204** , 751–758. (10.1242/jeb.204.4.751) 11171357

[43] Ishay JS , Rosenzweig E , Rosenzweig O , Berke S . 1989 Geotropic sensitivity of hornets. Adv. Space Res. **9** , 147–155. (10.1016/0273-1177(89)90069-0) 11537328

[44] Wyneken J . 2017 Sea turtle locomotion: mechanisms, behavior, and energetics. In The biology of sea turtles, pp. 165–198, vol. 1. Boca Raton, FL: CRC Press.

[45] Wang JH , Jackson JK , Lohmann KJ . 1998 Perception of wave surge motion by hatchling sea turtles. J. Exp. Mar. Biol. Ecol. **229** , 177–186. (10.1016/S0022-0981(98)00049-5)

[46] van der Geest N , Garcia L , Borret F , Nates R , Gonzalez A . 2023 Soft-robotic green sea turtle (Chelonia mydas) developed to replace animal experimentation provides new insight into their propulsive strategies. Sci. Rep. **13** , 11983. (10.1038/s41598-023-37904-5) 37491547 PMC10368674

[47] Pankaew K , Milton SL . 2017 The effects of extended crawling on the physiology and swim performance of loggerhead and green sea turtle hatchlings. J. Exp. Biol. **221** , jeb165225. (10.1242/jeb.165225) 29122949

[48] Pereira CM , Booth DT , Bradley AJ , Limpus CJ . 2013 Blood concentrations of lactate, glucose and corticosterone in dispersing hatchling sea turtles. Biol. Open **2** , 63–67. (10.1242/bio.20123046) 23336077 PMC3545269

[49] Mrosovsky N . 1968 Nocturnal emergence of hatchling sea turtles: control by thermal inhibition of activity. Nature **220** , 1338–1339. (10.1038/2201338a0) 5701356

[50] Segura LN , Cajade R . 2010 The effects of sand temperature on pre-emergent green sea turtle hatchlings. Herpetol. Conserv. Biol. **5** , 196–206.

[51] Desbiez ALJ , Massocato GF , Kluyber D , Santos RCF . 2018 Unraveling the cryptic life of the southern naked-tailed armadillo, Cabassous unicinctus squamicaudis (Lund, 1845), in a neotropical wetland: home range, activity pattern, burrow use and reproductive behaviour. Mamm. Biol. **91** , 95–103. (10.1016/j.mambio.2018.02.006)

[52] Booth DT . 2006 Influence of incubation temperature on hatchling phenotype in reptiles. Physiol. Biochem. Zool. Ecol. Evol. Approaches. **79** , 274–281. (10.1086/499988) 16555187

[53] Steenacker M , Tanabe LK , Rusli MU , Fournier D . 2023 The influence of incubation duration and clutch relocation on hatchling morphology and locomotor performances of green turtle (Chelonia mydas). J. Exp. Mar. Biol. Ecol. **569** , 151954. (10.1016/j.jembe.2023.151954)

[54] Saito T , Wada M , Fujimoto R , Kobayashi S , Kumazawa Y . 2019 Effects of sand type on hatch, emergence, and locomotor performance in loggerhead turtle hatchlings. J. Exp. Mar. Biol. Ecol. **511** , 54–59. (10.1016/j.jembe.2018.10.008)

[55] Najwa-Sawawi S , Azman NM , Rusli MU , Ahmad A , Fahmi-Ahmad M , Fadzly N . 2021 How deep is deep enough? Analysis of sea turtle eggs nest relocation procedure at Chagar Hutang Turtle Sanctuary. Saudi J. Biol. Sci. **28** , 5053–5060. (10.1016/j.sjbs.2021.05.021) 34466082 PMC8381072

[56] Dor D , Booth D , Schwanz LE . 2024 Data for: Swimming through sand: using accelerometers to observe the cryptic, pre-emergence life-stage of sea turtle hatchlings. Dryad Digital Repository. (10.5061/dryad.c866t1gdd)39353555

[57] Dor D , Booth D , Schwanz LE . 2024 Data from: Swimming through sand: using accelerometers to observe the cryptic, pre-emergence life-stage of sea turtle hatchlings. Figshare. (10.6084/m9.figshare.c.7468057)39353555

